# The spatial distribution of *cis *regulatory elements in yeast promoters and its implications for transcriptional regulation

**DOI:** 10.1186/1471-2164-11-581

**Published:** 2010-10-19

**Authors:** Zhenguo Lin, Wei-Sheng Wu, Han Liang, Yong Woo, Wen-Hsiung Li

**Affiliations:** 1 Department of Ecology and Evolution, University of Chicago, 1101 East 57th Street, Chicago, IL 60637, USA; 2 Department of Electrical Engineering, National Cheng Kung University, Tainan, Taiwan; 3 Biodiversity Research Center and Genomics Research Center, Academia Sinica, Taipei, Taiwan; 4 Department of Bioinformatics and Computational Biology, University of Texas M. D. Anderson Cancer Center, Houston, TX 77030, USA

## Abstract

**Background:**

How the transcription factor binding sites (TFBSs) are distributed in the promoter region have implications for gene regulation. Previous studies used the translation start codon as the reference point to infer the TFBS distribution. However, it is biologically more relevant to use the transcription start site (TSS) as the reference point. In this study, we reexamined the spatial distribution of TFBSs, investigated various promoter features that may affect the distribution, and studied the effect of TFBS distribution on transcriptional regulation.

**Results:**

We found a sharp peak for the distribution of TFBSs at ~115 bp upstream of the TSS, but no clear peak when the translation start codon was used as the reference point. Our analysis of sequence variation data among 63 yeast strains revealed very low deletion polymorphisms in the region between the distribution peak and the TSS, suggesting that the distances between TFBSs and the TSS have been selectively constrained in evolution. As in previous studies, we found that the nucleosome occupancy and the presence/absence of TATA-box in the promoter region affect the TFBS distribution pattern. In addition, we found that there exists a correlation between the 5'UTR length and the TFBS distribution pattern and we showed that the TFBS distribution pattern affects gene transcription level and plasticity.

**Conclusions:**

The spatial distribution of TFBSs obtained using the TSS as the reference point shows a much sharper peak than does the distribution obtained using the translation start codon as the reference point. The TFBS distribution pattern is affected by nucleosome occupancy and presence of TATA-box and it affects the transcription level and transcription plasticity of the gene.

## Background

The binding of gene-specific transcription factors (TFs) to the TF binding sites (TFBSs) in the promoter region is required for the initiation of gene transcription. In the last decade, extensive efforts have been made to identify TFBSs in *Saccharomyces cerevisiae *using both experimental and computational approaches [1-9]. These data are useful for studying the spatial distribution of TFBSs on yeast promoters. This distribution may be used to address questions on gene regulation such as whether there exists a "preferred zone" in the promoter for TF binding and how strongly the position of a TFBS affects the transcriptional output of a gene.

In *S. cerevisiae*, several studies have revealed that TFBSs are not uniformly distributed over the promoter region [2-4,10,11]. For example, using computational tools to search for over-represented binding motifs in various groups of genes in *S. cerevisiae*, Hughes et al. [4] found that TFBSs are highly enriched in the region approximately between 50 and 150 bp upstream of the translation start site. Similarly, Harbison et al. [3] found from ChIP-chip data that 74% of the TFBSs are located between 100 and 500 bp upstream of the translation start site. These studies have provided a rough spatial distribution of TFBSs. However, improvements can be made. First, since the transcription of a gene into mRNA begins at the transcription start site (TSS), it is biologically more relevant to study the TFBS distribution relative to the TSS than to the translation start codon. In this study we have used the TSS as the reference point, taking advantage of the high-resolution TSS data recently generated by RNA-seq [12]. Second, in previous studies the distribution was inferred using the number of TFBSs at each site of the promoter region, but the promoter length varies greatly among genes [13]. For example, there are 4977 sequences at position -200 bp (i.e., 200 bp upstream of the translation start site) but only 2494 sequences at position -400 bp (Yeast Genome Database). Clearly, the number of TFBSs at a site should be normalized by the number of promoter sequences that cover that site.

Two factors have been known to affect the distribution of TFBSs in eukaryotes: nucleosome positioning and presence of TATA-box [14-17]. As the primary building block of eukaryotic chromatins, a nucleosome is formed by tightly wrapping ~147 bp of DNA around a histone octamer [18]. The presence of nucleosomes is a major barrier for direct TF-TFBS interactions. Studies on nucleosome positioning have revealed that many yeast genes contain a nucleosome free region (NFR) in their promoters, which exposes TFBSs to TFs [14,15,17]. TFBSs are mainly located in the NFR in NFR-containing promoters but are more evenly distributed in NFR-less promoters, suggesting a significant effect of nucleosome positioning on TFBS distribution [15,19]. The TATA box is usually located ~40-120 bp upstream of the TSS in many yeast genes and facilitates in directing RNA polymerase II to the downstream TSS [20]. In *S. cerevisiae*, ~20% of the genes contain a TATA-box in their promoter regions [21]. The TATA box-containing genes and TATA box-less genes are known to use different transcription initiation pathways [21-23]. It has been shown that promoters with a TATA box have distinct structure features, such as higher nucleosome occupancy and higher DNA bendability near the TSS than do TATA box-less promoters, which may affect the TFBS distribution [16,19,24]. In this study, we re-evaluated the effects of nucleosome positioning and the presence/absence of TATA-box on the distribution patterns of TFBS inferred by our improved methods. In addition, we also studied whether there is a relationship between TFBS distribution and the length of the 5' untranslated region (5'UTR). Although the average length of 5'UTRs is rather constant among diverse organisms (100 - 200 bp), the length substantially varies among genes in the same genome [12,25,26]. It is not known if this variation in 5'UTR length has any relationship with the TFBS distribution and other promoter structural features, although it is well established that 5'UTR plays important roles in post-transcriptional regulation, including modulation of the mRNA subcellular localization, mRNA stability, transport of mRNAs out of the nucleus and translation efficiency [27-29]. Finally, we assessed the effects of the TFBS distribution on the output of gene transcription, which can be measured in two ways: (i) transcription abundance (level) under a given condition and (ii) transcriptional plasticity, which is defined as the capacity for a gene to change its transcriptional level under different conditions.

## Results

### A TFBS distribution peak at ~115 bp upstream of the TSS

We defined the promoter region of a gene as the intergenic region of the gene from its translation start codon to the coding boundary of the nearest upstream gene. We used the TSS as the reference point to infer the spatial distribution of TFBSs by measuring the frequency of TFBSs at each position in the promoter. For comparison, we also obtained a distribution using the translation start codon as the reference point. Since the total number and content of TFBSs strongly depend on the criteria for defining TFBSs, we analyzed four different sets of TFBS data to ensure the robustness of our conclusions (see Methods).

**Figure 1:**
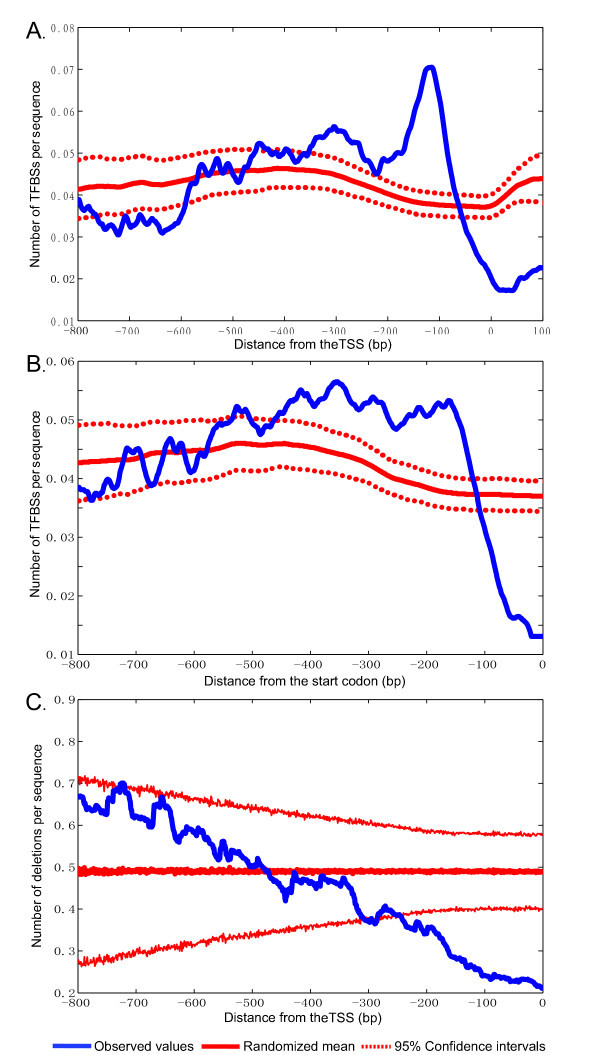
**The distribution of TFBSs in yeast promoters**. **(A)** The frequency distribution of the distances from the TFBSs to the transcription starting site (TSS) in genes. This figure shows the spatial distribution of TFBSs in the promoters of 4369 genes (using TFBS dataset IV). The value at each position relative to the TSS is a moving average of a window of 41 bp. The distribution has a very sharp peak ~115 bp upstream of the TSS and the TFBSs are strongly concentrated in the ~ 100 bp region from 180 to 80 bp upstream of the TSS. The blue solid line represents observed values; The red solid and dotted lines represent the mean of randomization and 95% confidence intervals for 1000 randomized tests **(B)** No sharp peak was found in the frequency distribution of TFBSs relative to the translation start codon. This figure was generated using the same data as in **(A)** except that the translation start codon instead of the TSS was used as the reference point. **(C)** The frequency of deletion polymorphisms in the TSS-proximal region is the lowest in the promoter region and is significant lower than random expectation. This figure was generated using the same data as in **(A)**.

With the TSS as the reference point, we found TFBSs to be highly enriched from ~80 to 200 bp upstream of the TSS with a sharp peak at -115 bp; the four different TFBS datasets yield highly consistent overall patterns (Figure 1A and Additional file 1). The frequencies of TFBSs at the peak are significantly higher than the average randomized distribution of TFBSs, indicating a strong positioning bias of TFBSs relative to the TSS (Figure 1A). In contrast, with the start codon as the reference point, only a plateau, but no sharp peak, is observed (Figure 1B and Additional file 1). Similar TFBS distributions were obtained when we excluded bidirectional promoters, each of which is a promoter region shared by two divergently transcribed adjacent genes (Additional file 1), highlighting the robustness of our results. To statistically evaluate if the TFBSs have more biased distribution inferred using the TSS as the reference point than does the distribution obtained using the translation start codon as the reference point, we calculated the localization bias of the peak which is defined as the ratio of the maximal value of the TFBS frequencies divided by the average value of the TFBS frequencies (we used 1000 bootstrap pseudoreplicates; see Methods). We found that localization bias of the peak inferred by the TSS (mean ratio = 1.69 ± 0.07) is significant higher than that inferred by the start codon (mean ratio = 1.35 ± 0.05, p-value < 10^-15 ^, t-test) supporting the existence of a preferred range of distances from TFBSs to the TSS.

To ensure the robustness of the above results, we also inferred the distribution of TFBSs retrieved from SwissRegulon [30], which contained 14001 high confidence binding sites for 72 TFs with a posterior probability > 0.5 (see Methods). Similar to the above results, the TFBSs are highly enriched in the region of 80-200 bp upstream of the TSS, and the distribution of TFBSs is less biased when the start codon is used as the reference (Additional file 2). Based on 1000 bootstraps, the localization bias of the peak of TFBS distribution inferred by the TSS (mean ratio = 2.6764 ± 0.0708) is significant higher than that inferred by the start codon (mean ratio = 1.9391 ± 0.0610) (p-value <10^-15^, t-test). Therefore, our analysis appearsto be robust.

Interestingly, the frequency of TFBSs immediately downstream of the peak, including the 5'UTR region, is significantly lower than random expectation and the frequency in any other intergenic regions and random expectation, revealing a TFBS-depleted region from -80 bp to the 5'UTR (Figure 1A). This region roughly corresponds to the location of the core promoter [31]. The core promoter contains DNA elements that directly interact with the general transcription machinery and can extend 35 bp upstream or downstream of the TSS [31]. This TFBS-depleted region can be explained by the selection against potential spatial competition between TF binding and the general transcription complex. The depletion of TFBS in the 5'UTR region indicates that TFBSs strongly prefer the location upstream to the region where the general transcription complex binds.

### Strong selective constraints on the distances between TSS and TFBSs

If the distance between a TFBS and the TSS is important for the TF function, DNA insertion and deletion events in this region should tend to be selectively disadvantageous. To test this hypothesis, we estimated the frequency of deletion polymorphisms using the genome-wide polymorphism data of 63 *S. cerevisiae *strains from diverse ecological niches and geographic locations [32]. For all promoters with an identified TSS location, we computed the average occurrence of deletion events per sequence at each position relative to the TSS (see Methods). Figure 1C shows that the frequency of deletion polymorphisms monotonically decreases as the distance to the TSS decreases. In addition, the frequency of deletion polymorphisms in the region from ~-200 bp to TSS is significantly lower than the random expectation. Fewer deletion polymorphisms in the TSS proximal region suggest a lower level of tolerance of deletion mutations. Our data shows that most TFBSs are located within the first 200 bp upstream of the TSS and that the number of TFBSs decreases as the distance to the TSS increases. Therefore, the frequency of deletion polymorphisms is negatively correlated with TFBS occurrence, suggesting that the distance between a TFBS and the TSS is under purifying selection. In contrast, no significant variation in SNP frequency is detected among different promoter regions (Additional file 3), probably because the occurrence of SNP does not change the TFBS-TSS distance.

### Effects of nucleosome occupancy and presence/absence of TATA-box on the TFBS distribution

**Figure 2:**
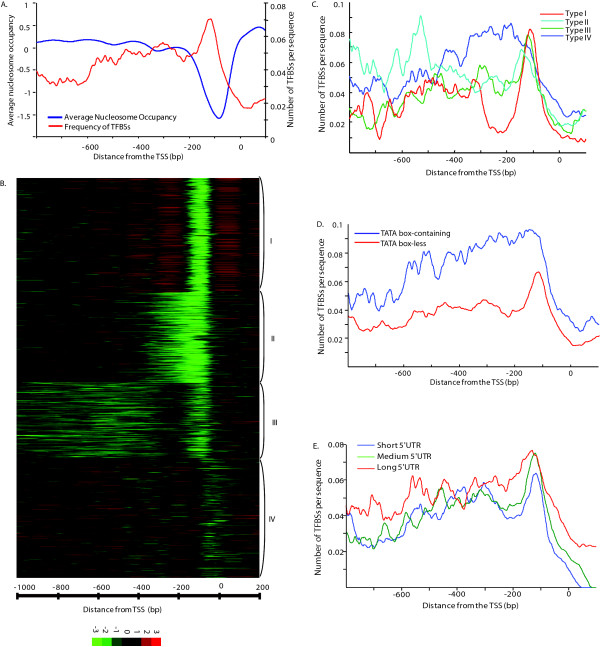
**The effects of promoter architecture features on the TFBS distribution**. **(A) **Strong negative correlation between the TFBS frequency and the genome-wide average of nucleosome occupancy in the ~200 bp region upstream of the TSS. The intergenic regions were aligned with reference to TSS. (**B) **The yeast genes were clustered into four groups by k-means clustering based on a 1201 bp region surrounding the TSS. **(C) **The frequency distributions of TFBSs relative to the TSS in four clusters of genes with diverse nucleosome occupancy patterns. The figure is presented in the same way as Figure 1A. **(D) **The TFBS distribution differs between TATA box-containing and TATA box-less genes. The TATA box-containing genes have more broadly distributed TFBSs and higher TFBS frequency in the promoter region. **(E) **The distributions of TFBSs relative to the TSS in three categories of 5'UTR length: short, medium and long. The long 5'UTR genes have more broadly distributed TFBSs and a higher TFBS frequency in the promoter region.

We calculated the average nucleosome occupancy in the 1201 bp region surrounding the TSS for all yeast genes using the recently released nucleosome occupancy data by Kaplan et al. [17] (see Methods). The genome average nucleosome occupancy reveals a NFR in the ~200 bp region immediately upstream of the TSS (Figure 2A), which is consistent with a previous study based on different nucleosome data [15]. As shown in Figure 2A, there is a negative correlation between TFBS density and nucleosome occupancy. The peak of the TFBS distribution matches the valley of nucleosome occupancy, indicating that TFBSs are predominantly enriched in the NFR. The negative correlation between the TFBS distribution and nucleosome occupancy has been observed on individual TFs, such as ABF1, REB1, MBP1 and RSC3 [15,33]. The consistency between the genome-wide scale TFBS distribution and the distribution for individual genes suggests that the presence of NFR is an important factor for the uneven spatial distribution of TFBSs in *S. cerevisiae*.

To further investigate how low nucleosome occupancy surrounding the TSS affects the distribution of TFBSs, we used the k-means clustering algorithm to classify all genes into four clusters based on their nucleosome positioning patterns in the promoter region [15] (see Methods). The four clusters (Type I to Type IV) contain 1,181, 927, 781 and 1,232 promoters, respectively (Figure 2B). Type I promoters have the most conspicuous 200 bp NFR with well defined boundaries, whereas Type IV promoters are mostly occupied by nucleosomes. Similar to the Type I promoters, Type II and III promoters also contain a nucleosome-depleted region upstream of the TSS, but their NFRs lack a well positioned upstream nucleosome (-1 nucleosome). Interestingly, these four types of promoters display distinct TFBS distribution patterns (Figure 2C). Type I promoters have the most uneven TFBS distribution among all groups, with a very sharp peak at ~115 bp upstream of the TSS; whereas Type IV promoters show no sharp peak and are broadly distributed between -100 and -500 bp (Figure 2C). The presence of a NFR close to the TSS results in the enrichment of TFBSs within the NFR. Although the TFBSs of Type IV promoters are relatively broadly distributed in the promoter region, the frequencies of TFBSs are significantly higher than in other types of promoters at most positions. On average, more TFBSs are present in a Type IV promoter than a promoter with a NFR, suggesting a more sophisticated regulation for genes with Type IV promoters. Tirosh et al. [19] identified two extreme classes of promoters based on nucleosome occupancy: depleted proximal-nucleosome (DPN) and occupied proximal-nucleosome (OPN). They showed that TFBSs are strongly enriched in the NFR of DPN promoters, but are more evenly distributed throughout OPN promoters. DPN and OPN genes are similar to the Type I and IV genes, respectively, regarding the nucleosome occupancy pattern and the TFBS distribution. However, our normalized distribution of TFBSs on the Type IV promoters provides extra information: TFBSs of Type IV promoters are not only more uniformly distributed, but with a significant higher density of TFBSs compared to Type I promoters. Therefore, our results suggest that although the presence of a nucleosome may reduce the accessibility of TFBSs for the TFs, it does not necessarily reduce the occurrence of TFBSs. Indeed, more TFBSs are found in nucleosome-occupied promoters, suggesting that genes with nucleosome-occupied promoters tend to be regulated by more TFs.

We compared the TFBS distributions between 1,097 TATA box-containing and 5,649 TATA box-less genes. As shown in Figure 2D, the TFBSs in TATA box-less genes show a sharp peak upstream of the TSS, whereas a high plateau instead of a clear peak of the TFBS distribution is observed in TATA box-containing genes. In addition, higher frequencies of TFBSs are observed in TATA box-containing genes than in TATA box-less genes at all positions, indicating that TATA box-containing promoters tend to contain more TFBSs. Therefore, the TATA box-containing genes have a similar TFBS distribution pattern to Type IV genes, suggesting that these genes may be under more intricate regulation.

### Association of different 5'UTR lengths with distinct TFBS distributions and nucleosome occupancy patterns

The observation of a higher variation in 5'UTR length within species than between species motivated us to investigate its relationship with promoter structures. To study this question, we divided all *S. cerevisiae *genes into three groups according to the lengths of 5'UTR: short (x < 39 bp, 1460 genes), medium (39 ≤ x < 81, 1471 genes) and long (x ≥ 81 bp, 1483 genes). Distinct TFBS distributions are observed among the three groups. As shown in Figure 2E, the long 5'UTR genes have the highest frequency of TFBSs and most broadly distributed TFBSs in the promoter region. Especially, long 5'UTR genes contain a significant higher frequency of TFBSs in the 5'UTR region than short 5'UTR genes (Figure 2E). In contrast, the TFBSs of short 5'UTR genes are enriched in a short region of 80-180 bp upstream of the TSS, showing a negative correlation between 5'UTR length and uneven distribution of TFBSs. We repeated these analyses by dividing all genes in to four groups of 5'UTR length and observed a similar trend.

**Figure 3:**
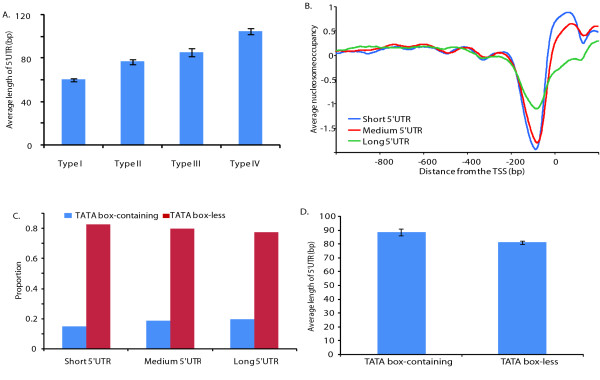
**Intrinsic correlations among nucleosome positioning, presence/absence of TATA box and 5'UTR length**. **(A) **Average 5'UTR lengths among the four gene groups clustered by k-mean clustering based on their nucleosome occupancy patterns. The mean value of each group is indicated by a bar and the error bars indicate one standard error. (**B) **The nucleosome occupancy in the 1201 bp window surrounding the TSS in the three groups of genes with different 5'UTR lengths. The TSS proximal region of long 5'UTR genes has the highest nucleosome occupancy. **(C) **The proportion of TATA box-containing genes and TATA box-less genes in each 5'UTR length group of genes. The long 5'UTR genes have the highest proportion of TATA box-containing genes, but the lowest proportion of TATA box-less genes. **(D) **TATA box-containing genes have slightly longer 5'UTRs than do TATA box-less genes.

To obtain further information about the relationship between 5'UTR length and the distribution of TFBSs, we studied the correlations of 5'UTR length with nucleosome positioning and the presence/absence of TATA box. First, we found a significant positive correlation between 5'UTR length and the nucleosome occupancy level near the TSS (Figure 3A and 3B). Specifically, the average 5' UTR length of Type IV genes is nearly as twice long as that of Type I genes (103.9 bp vs 60 bp, p < 10^-40^, t-test), while the Type II and III genes have intermediate 5' UTR lengths (Figure 3A). Second, long 5'UTR genes tend to have much higher nucleosome occupancy near the TSS compared with short 5'UTR genes (Figure 3B). In addition, we also observed a positive correlation between the 5' UTR length and presence of TATA-box (Figure 3C and 3D). TATA box-containing genes are enriched in long 5'UTR genes (p = 0.0002, Chi-square test), and TATA box-less genes tend to have short 5'UTRs (p = 0.008) (Figure 3C). Therefore, TATA box-containing genes have, on average, longer 5'UTRs than TATA box-less genes (p = 0.006, t-test) (Figure 3D). We speculate that promoter structural features, such as nucleosome occupancy and presence/absence of TATA box, affect the 5'UTR length, because they play a more important role in transcription regulation than does the 5'UTR length. However, we cannot rule out the possibility that the reverse is true.

### Effects of promoter features on gene transcription profiles

**Figure 4:**
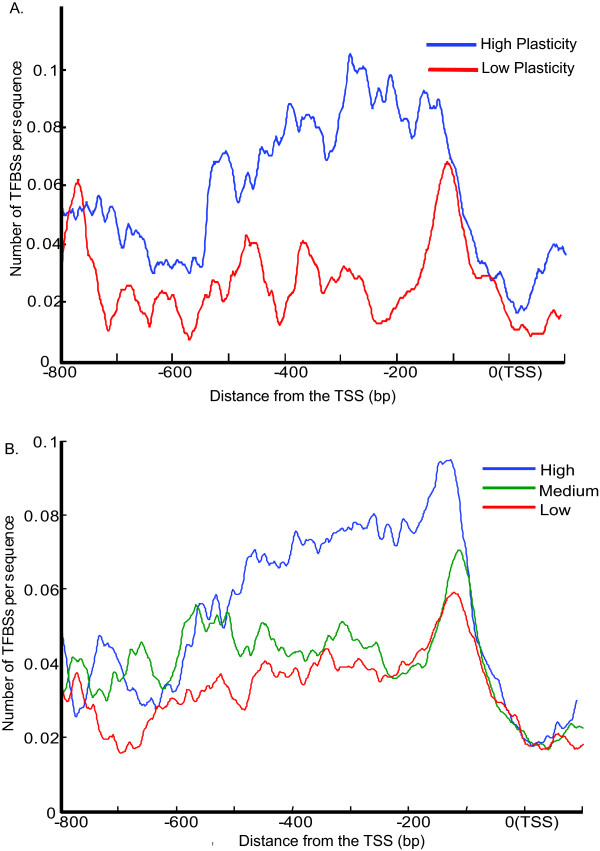
**The distribution patterns of TFBSs among genes with different expression profiles**. **(A) **High expression plasticity genes have distinct TFBS distribution patterns compared to the low plasticity genes. (**B) **The density of TFBSs has a positive correlation with gene expression level under rich media.

To test if the TFBS distribution pattern affects gene transcriptional regulation, we infer the TFBS distribution patterns for genes with different transcriptional plasticity values and transcriptional abundances (levels). We calculated the transcriptional plasticity value for each gene from over 1000 profiles of microarray data (see Methods) and ranked all genes by their transcriptional plasticity values. The TFBS distribution patterns were computed for 500 genes with the highest plasticity and for 500 genes with the lowest plasticity, respectively. As shown in Figure 4A, the TFBSs of low plasticity genes form a sharp peak around 100 bp upstream of the TSS, showing a highly uneven TFBS distribution. In contrast, the TFBSs of high plasticity genes are broadly distributed in a 400 bp region, from -500 to -100 bp upstream of the TSS. More importantly, the density of TFBSs in this region is even higher for high plasticity genes. This result suggests that the large number of TFBSs in the promoter is an important factor for the plasticity of gene expression.

We also studied the TFBS distribution patterns for genes with different expression levels under normal growth conditions. Nagalakshmi et al. [12] used RNA-seq data to divide yeast genes into three expression groups: high, medium and low. We found that the TFBSs of the three groups are not very different with regard to the distribution shape in the promoter region (Figure 4B). However, significant differences can be seen in terms of the frequency of TFBSs. Indeed, the overall density of TFBSs is positively correlated with the expression abundance, suggesting that genes with more TFBSs in their promoter regions tend to have a higher expression level.

## Discussion

Our study reveals that TFBSs are highly enriched in a narrow region ~115 bp upstream of the TSS, whereas no clear distribution peak is observed when the translation start codon is used as the reference point (Figure1 and S1). This result differs from the observation of previous studies [3,4]. The difference arose because different methods were used to infer the distribution of TFBSs. As mentioned above, previous studies did not normalize the number of TFBSs observed at a position by the number of promoters that covered that position. One can easily imagine that the proximal regions of the translation start site have the largest number of sequences, whereas more upstream regions have fewer sequences. Our revised distribution of TFBSs suggests that the distance between TFBSs and TSS is important for transcriptional regulation. The distribution pattern of TFBSs can be valuable for binding motif prediction because candidate TFBSs at different locations should be given different weights. When genome-wide TSS and TFBS data becomes available for other closely related species of *S. cerevisiae*, it will be interesting to see whether the spatial distribution of TFBSs have been conserved in evolution.

Our observation that both nucleosome positioning and presence/absence of TATA box affect the distribution of TFBSs is consistent with previous studies [15,16,19,21]. The DNA sequence in the NFR is exposed and therefore accessible for TFs, because no chromatin remodelling and no DNA unwrapping are required for TF-TFBS interaction, which is an advantageous structure for constantly transcribed genes. Therefore, it is believed that there are more TFBSs in the NFR than in a nucleosome occupied region [15]. This is true in general, but the nucleosome occupancy pattern in the promoter region does not strongly affect the total number of TFBSs in the promoter. In fact, the TFBS density for nucleosome-occupied promoters (e.g. Type IV) tends to be even higher than the peak of NFR-containing promoters (e.g. Type I genes, Figure 2C). More TFBSs are also observed in the TATA-box containing genes (Figure 2D).

It has been shown that the genes with nucleosome occupied promoters and TATA-box containing genes have higher expression variability under different conditions, compared to the other types of genes [16,19]. Our Gene Ontology analysis reveals that Type IV genes are significantly enriched in oxidation reduction and response to various stimuli (Additional file 4), similar to the TATA box-containing genes [21]. Stress-response genes need to be finely regulated, and so their expression level can be dramatically changed under different conditions. In addition, genes with high expression plasticity also contain more TFBSs in the promoter region (Figure 4A). Therefore, the presence of more TFBSs in the promoters of these genes provides higher flexibility for different TFBS combinations under different conditions.

## Conclusions

Our study showed that the spatial distribution of TFBSs has a sharp peak at 115 bp upstream of the TSS, which is inside the nucleosome-depleted region. In contrast, no clear peak of the TFBS distribution was observed using the translation start codon as the reference point. The frequency of deletion polymorphisms monotonically decreases as the distance to TSS decreases, while no significant variation in nucleotide polymorphism frequency was observed along the promoter region, suggesting that the distance between TFBSs and the TSS is functionally constrained. Our study further indicated that the TFBS distribution pattern is affected by nucleosome occupancy and presence of TATA-box and that the distribution pattern affects the transcription level and transcription plasticity of the gene.

## Methods

### Data sources

The TFBS locations for 117 TFs were determined according to the motif-discovery algorithm of MacIsaac *et al. *[6], which is based on genome-wide Chip-chip data [3] and sequence conservation among species. We used four TFBS data sets that are defined in MacIsaac et al. by using different combinations of TF binding confidence of the ChIP-chip data and TFBS conservation level: (I) a stringent TF binding criterion (p < 0.001) and strong evolutionary conservation of TFBS (conserved in at least three of the four yeast species: *S. cerevisiae*, *S. paradoxus*, *S. mikatae *and *S. bayanus*); (II) a stringent binding criterion (p < 0.001) and moderate conservation (conserved in at least two yeast species); (III) a moderate binding criterion (p < 0.005) and strong conservation (conserved in at least three yeast species), and (IV) a moderate binding criterion (p < 0.005) and moderate conservation (conserved in at least two yeast species). Dataset (I) represents the most stringent one, whereas dataset (IV) is the most relaxed and contain the largest number of predicted TFBSs.

To ensure the robustness of our analysis, we also used the TFBS dataset from SwissRegulon database [30]. This TFBS dataset was produced using the MotEvo TFBS prediction algorithm, which operates on multiple alignments of orthologous intergenic regions from 5 closely related yeast species in combination with a collection of experimentally known binding sites from the yeast promoter database SCPD, and ChIP-chip binding data. We retrieved 14001 high confidence binding sites for 72 TFs with a posterior probability > 0.5 from this database and used them to inferred TFBS distribution relative to the TSS and the start codon, respectively.

The genomic coordinates of TSS, RNA expression level, and 5'UTR length were obtained from Nagalakshmi *et al. *[12], in which a high-resolution transcriptome of the yeast genome was generated by a high-throughput RNA-seq method. The lists of TATA box-containing genes and TATA box-less genes were obtained from Basehoar et al. [21]. The nucleosome occupancy data were determined by deep sequencing under three different *in vivo *conditions, YPD, galactose and ethanol [17]. The nucleosome occupancy data obtained under YPD were used in this study, because the TSS data was also obtained under this condition. The transcriptional or expression plasticity was estimated as the average of the squared log2 expression ratio from over 1000 microarray experiments, which reflects the capacity for a gene to change its transcriptional level under different conditions [34]. The gene lists of all defined promoter structural groups are provided in Additional file 5. The data (TFBS, TSS, TATA box, nucleosome occupancy) used in this paper were organized in an online browser at http://zoro.ee.ncku.edu.tw/ypa/.

### Determining the distribution of TFBSs

We constructed the distribution of TFBSs at a position by dividing the number of TFBSs at that position with the number of promoter sequences that cover that position, rather than just counting the total number of TFBSs at the position, because the numbers of promoter sequences at different positions vary greatly. Let G be a set of genes of interest. Let x be the position relative to TSS. For each x in the promoter region of a gene in G, we checked whether a TFBS is located at that position or not by using the TFBS data in MacIsaac et al. [6]. The same process was applied to all genes in G. Then we counted the total number, n(x), of TFBSs located at position x for all genes in G. Finally, the TFBS frequency d(x) at position x was obtained by dividing n(x) by the total number of promoter regions r(x) that contain position x. The frequency of TFBSs at each position was smoothed by a moving average of a window of size 41 bp. The binding sites in each promoter region of the 4369 genes were redistributed randomly and independently in the promoter region to generated a "randomized" TFBS dataset which was then used to infer the random expectation of the TFBS frequency by following the approach in [3]. The randomization process was repeated 1000 times to obtain 95% confidence intervals of random expectation values at each position. The observed frequency of TFBSs relative to the start codon and its randomized expectation were inferred using the same method.

To determine if TSS-based TFBS distribution is more biased than the distribution based on the start codon, we compared the localization bias of the peak which is defined as the ratio of the maximal value of the TFBS frequencies divided by the average value of the TFBS frequencies [19]. We used bootstrapping to generate 1000 ratio values. For each bootstrap pseudoreplicates, 4369 resamples were obtained by random sampling with replacement from the original gene set used in this study. Then these 4369 resamples were used to calculate the TFBS frequency for each position in the promoter region. We obtained 1000 peak-to-average ratios for TSS-inferred and the start codon-inferred TFBS distributions, respectively, and we conducted 2-sample t test to determine if there is a significantly different localization bias between the two distributions.

### Estimating the frequency of deletion polymorphisms and SNPs in promoter region

The SNP and deletion polymorphism data were obtained from Schacherer et al [32]. The frequency of deletion at each position relative to the TSS was calculated in an 800 bp region upstream of the TSS. If the length (*L*) between the TSS and coding region boundary of nearest upstream gene was > 800 bp, only the first 800 bp region was used. The actual length was used if *L *< 800 bp. We counted the total number of detected deletion events (*N*_*d*_) at each position relative to the TSS. The frequencies of deletion events (*F*_*d*_) were calculated through dividing *N*_*d *_by total number of sequences at each position. The frequencies of SNPs were also estimated for the same 800 bp region. The total number of SNPs at each position relative to the TSS was counted for all intergenic sequences using data from the same source [32]. The genome-wide average frequency of SNP was calculated through dividing the total number of SNPs by the total number of sequences at each position relative to the TSS and then smoothed by moving average of a window of 41 bp. We inferred the random expectation frequency of the deletion polymorphism and SNPs based on the randomized data which was generated by randomly shuffling the locations of deletion polymorphisms and SNPs in all intergenic regions. This process was repeated 1000 times to obtain the 95% confidence intervals of the random frequency of deletion polymorphisms and SNPs at each position.

### Yeast gene clustering, estimation of average nucleosome occupancy, and gene transcriptional plasticity and abundance

We retrieved nucleosome occupancy data of the 1,201 bp region surrounding TSS (-1000 to 200 bp) for the 4,556 genes with identified TSS locations [17]. The position of nucleosome occupancy in each gene was aligned relative to the TSS. The total nucleosome occupancy values (log2 mean) of each position in the 1201 bp region were calculated for all genes in a defined group. The average nucleosome occupancy per position was then calculated by dividing the number of genes in each group. Because nucleosome occupancy data are not available in some genomic regions, we only used promoters with at least 80% coverage of nucleosome occupancy data in the clustering of genes. In total, 4121 genes were used in k-means clustering with Cluster 3.0 using the Euclidean distance metric and 20 repetitions [35]. Clusters were visualized with Java Treeview [35]. The transcriptional plasticity for each gene was quantified as the average of the squared log2 expression ratio. We used the same method of transcriptional plasticity estimation that was described in Tirosh and Barkai [19]. We used the transcription abundance data from Nagalakshmi *et al. *[12] to evaluate the effects of promoter architecture on the transcription level, because the nucleosome occupancy and the TSS data used in this study were also measured during vegetative growth in rich media.

## Authors' contributions

ZL, WW, HL and WHL designed the study. ZL, WW and YW performed the data analysis. WHL, ZL, WW and HL wrote the paper. All authors read and approved the final manuscript.

## Supplementary Material

Additional file 1 **The spatial distribution of TFBSs in different TFBS datasets from MacIsaac et al.'s**. **(A)**. The overall distribution of TFBSs relative to the TSS. This figure shows distribution pattern based on four datasets from MacIsaac et al.'s including all genes in the yeast genome. **(B) **The overall distribution of TFBSs relative to the TSS. This figure shows distribution pattern based on four datasets excluding bidirectional promoters. **(C) **The overall distribution of TFBSs relative to the translation start codon. This figure shows the distribution pattern based on four datasets including all genes in the yeast genome. **(D) **The frequency distribution of TFBSs relative to the translation start codon. This figure shows distribution patterns based on four datasets excluding bidirectional promoters. Click here for file

Additional file 2 **The spatial distribution of TFBSs based on the SwissRegulon dataset**. **(A) **The distribution of TFBSs relative to the TSS based on ~14000 high confidence binding sites for 72 TFs with a posterior probability > 0.5 from SwissRegulon. **(B) **The distribution of TFBSs relative to the translation start codon based on the same dataset. Based on 1000 bootstraps, the peak-to-average ratio of TFBS distribution inferred by the TSS (mean ratio = 2.6764, std = 0.0708) is significant higher than that inferred by the start codon (mean ratio = 1.9391, std = 0.0610) with p-value <10^-15 ^, t test. Click here for file

Additional file 3 **Frequency of SNP in the 1201 bp region surrounding the TSS**. The frequency of SNP was calculated through averaging the total number of SNPs at a given position relative to the TSS by the number of sequences, which is shown in blue dots. The trend of SNP frequency is generated by moving averages of 41 bp window. No significant difference in the frequency of SNPs can be observed between different promoter regions. The solid cyan line represents moving average; the solid and dot red lines indicate, respectively, the mean and 95% confidence intervals for 1000 randomized tests Click here for file

Additional file 4 **The GO Term Finder results of the Type I-Type IV genes**. The significant GO terms (top 10 hits) shared among each group of Type I-Type IV genes from *S. cerevisiae *were found by using GOTERMFINDER (http://go.princeton.edu/cgi-bin/GOTermFinder). Only top 10 hits in each type of genes were shown in the table. Click here for file

Additional file 5 **A complete list of genes used in this study**. This table includes the lists of genes of each group of TATA box-containing, TATA box-less, Type I-Type IV and the data of 5'UTR length, transcriptional level (log2) and gene expression plasticity that were used in this study. Click here for file
