# Supplementary figures and images for: The spatial distribution of cis regulatory elements in yeast promoters and its implications for transcriptional regulation
Source: BMC Genomics. 2010 Oct 19;11:581. doi: 10.1186/1471-2164-11-581 (PMC3091728; doi:10.1186/1471-2164-11-581)

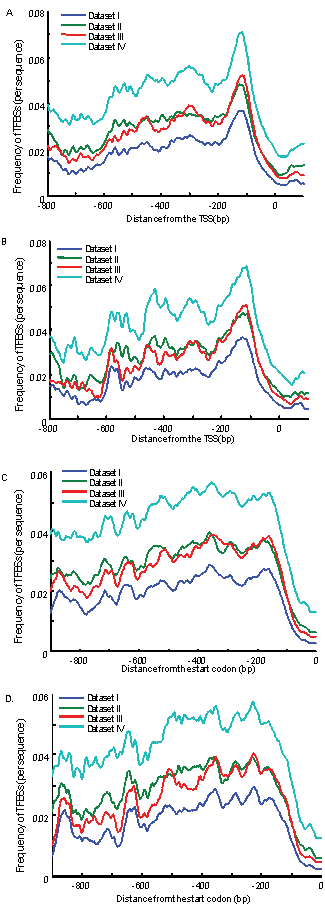

Supplement: Additional file 1 — The spatial distribution of TFBSs in different TFBS datasets from MacIsaac et al.'s. (A). The overall distribution of TFBSs relative to the TSS. This figure shows distribution pattern based on four datasets from MacIsaac et al.'s including all genes in the yeast genome. (B) The overall distribution of TFBSs relative to the TSS. This figure shows distribution pattern based on four datasets excluding bidirectional promoters. (C) The overall distribution of TFBSs relative to the translation start codon. This figure shows the distribution pattern based on four datasets including all genes in the yeast genome. (D) The frequency distribution of TFBSs relative to the translation start codon. This figure shows distribution patterns based on four datasets excluding bidirectional promoters. [file 1471-2164-11-581-S1.PNG]

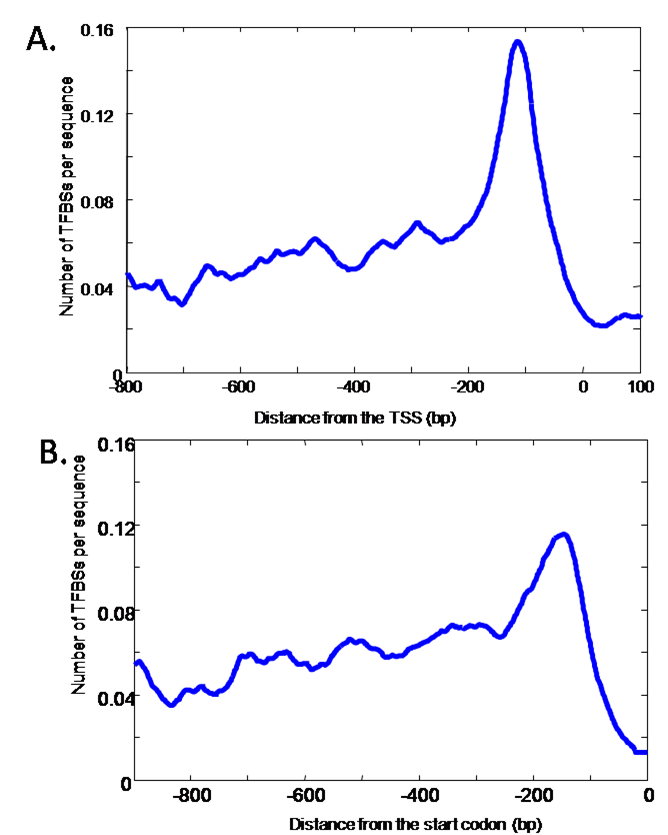

Supplement: Additional file 2 — The spatial distribution of TFBSs based on the SwissRegulon dataset. (A) The distribution of TFBSs relative to the TSS based on ~14000 high confidence binding sites for 72 TFs with a posterior probability > 0.5 from SwissRegulon. (B) The distribution of TFBSs relative to the translation start codon based on the same dataset. Based on 1000 bootstraps, the peak-to-average ratio of TFBS distribution inferred by the TSS (mean ratio = 2.6764, std = 0.0708) is significant higher than that inferred by the start codon (mean ratio = 1.9391, std = 0.0610) with p-value <10-15 , t test. [file 1471-2164-11-581-S2.PNG]

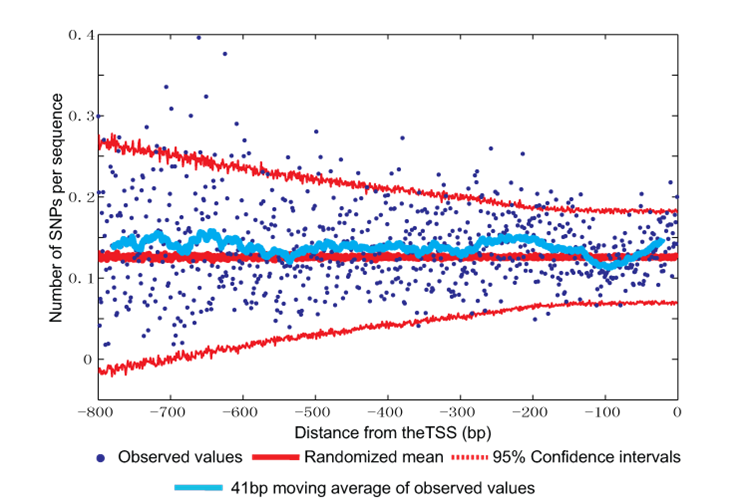

Supplement: Additional file 3 — Frequency of SNP in the 1201 bp region surrounding the TSS. The frequency of SNP was calculated through averaging the total number of SNPs at a given position relative to the TSS by the number of sequences, which is shown in blue dots. The trend of SNP frequency is generated by moving averages of 41 bp window. No significant difference in the frequency of SNPs can be observed between different promoter regions. The solid cyan line represents moving average; the solid and dot red lines indicate, respectively, the mean and 95% confidence intervals for 1000 randomized tests [file 1471-2164-11-581-S3.PNG]
